# Self‐Interfering Magnetic Dipole Transition Emission in Rare‐Earth Ions Doped Nanoparticles Modulated by Plasmonic Mirror Nanocavity

**DOI:** 10.1002/nap2.70065

**Published:** 2026-04-10

**Authors:** Chengxiang Gou, Bowen Kang, Wangze Li, Xiangtai Xi, Yuting Luo, Jiaqi Wang, Zhengkun Fu, Huan Chen, Zhenglong Zhang, Hairong Zheng

**Affiliations:** ^1^ School of Physics and Information Technology ShaanXi Normal University Xi'an China

**Keywords:** atomic force microscopy manipulation, branching ratio, electric dipole and magnetic transition emissions, local density of optical states, plasmonic mirror nanocavities, rare‐earth ions doped nanoparticles, self‐interference effects

## Abstract

In conventional photonics, light–matter interactions are predominantly governed by electric dipole (ED) transition. Although magnetic dipole (MD) transition emission is challenging to detect due to its intrinsic properties, it exhibited significant application potential in biological probes and quantum technologies. Eu^3+^ ions are considered an ideal system for investigating MD–ED transition emissions owing to their unique energy‐level structure as sensitive nanoscale probes for electromagnetic fields. In this paper, a mirror plasmonic nanocavity combining Au nanospheres (NSs) with an Au flake is applied to modulate both ED and MD emissions from Eu^3+^‐doped NaYF_4_ nanoparticles (NPs). The structure of the mirror nanocavity is systematically adjusted and precisely controlled through the nanoscale manipulation capability of atomic force microscopy (AFM). The emission of MD transition is enhanced as 3‐fold on Au flake through the construction of a monolayer Eu^3+^ ions doped NPs only by self‐interference effect. By coupled Au NSs and flake, the intensity of ED transition emissions was restored to 481 times its original magnitude through the utilization of localized surface plasmon resonance (LSPR) effects. A significant enhancement of Eu^3+^ ions luminescence and artificial modulation of emission ratios are achieved. This tunable platform benefits for detection and manipulation of light–matter interactions at nanoscale.

## Graphical abstract

The mirror‐type plasmonic nanocavity, composed of Au nanospheres (NSs) and an Au flake, is manipulated at the nanoscale via atomic force microscopy (AFM) to modulate both electric dipole (ED) and magnetic dipole (MD) emissions from Eu^3+^‐doped NaYF_4_ nanoparticles (NPs). A 481‐fold enhancement of ED transition emission and artificial modulation of Eu^3+^ ions emission ratios are achieved by utilizing localized surface plasmon resonance (LSPR) effects.

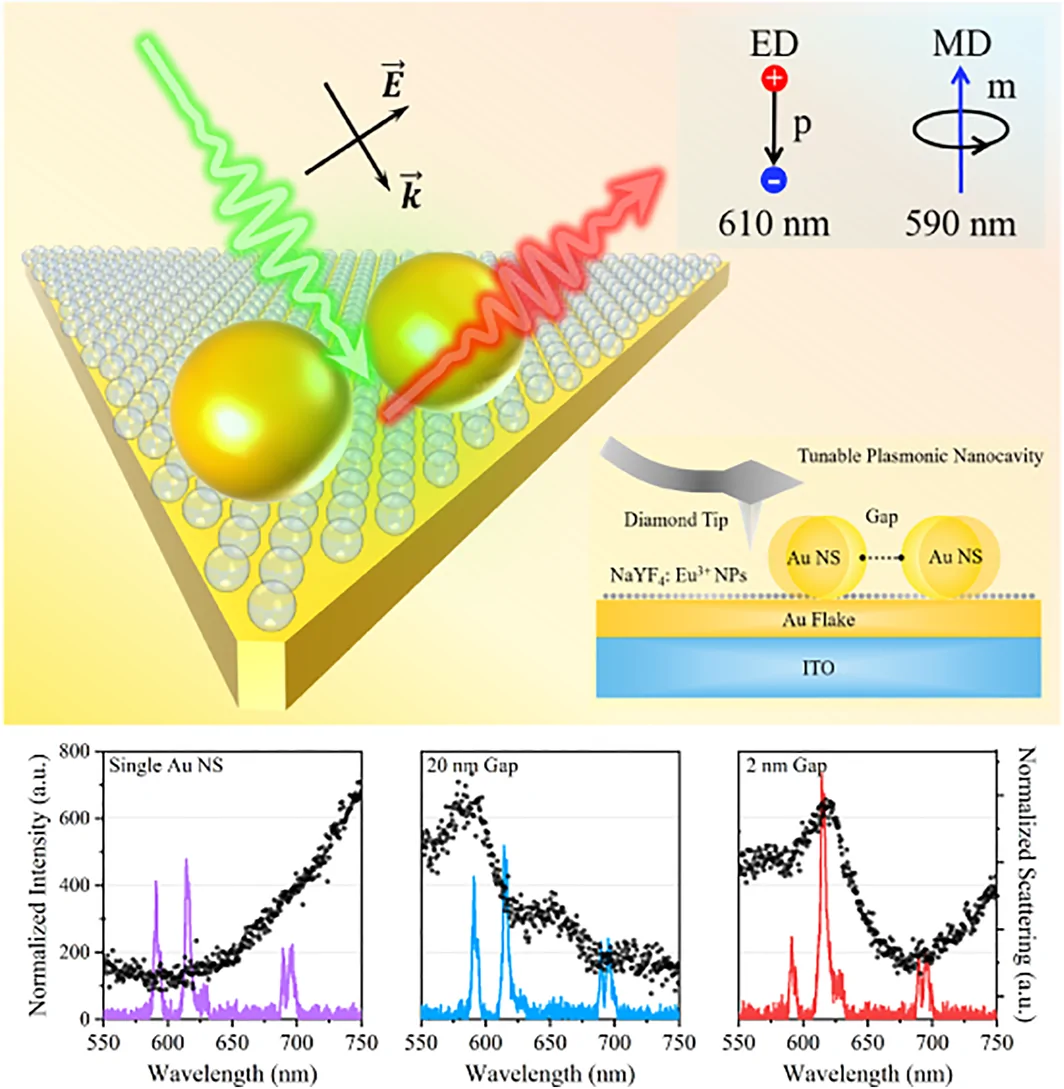

## 1 Introduction

Substantial research efforts in traditional photonics were devoted to controlling and enhancing the spontaneous emission process of electric dipole (ED) transitions [1, 2]. Across optical frequency ranges, the interactions between light and matter were predominantly dictated by the material’s response to an applied electric field [3]. It was widely accepted that electric fields were the primary mediators of light–matter interactions, whereas the Lorentz force generated by optical‐frequency magnetic fields was considered to exert a negligible influence [4, 5]. Due to the fine‐structure constant (*α* ≈ 1/137), the magnetic dipole (MD) transition emissions in optical materials were significantly weaker than ED transition emissions [6, 7]. Experimental efforts to detect magnetic fields at optical frequencies were severely hindered by an interaction strength with matter. Nevertheless, the ability to engineer a substantial enhancement in the emission rates of such weak sources was understood to hold considerable value for numerous applications, encompassing advanced displays, fluorescent biomarkers, and sources of quantum light. Furthermore, MD emitters were successfully implemented as effective substitutes for conventional near‐field probes in the spatial mapping of optical magnetic fields, applicable to laser beams, plasmonic nanostructures, optical antennas, and metamaterials. Consequently, the precise engineering of light–matter interactions mediated by MD transitions was confirmed to be of dual significance, encompassing both fundamental scientific inquiry and practical technological applications [8, 9, 10, 11, 12].

The significance of magnetic interactions was largely confined to materials with symmetry‐forbidden ED channels as exemplified by intraconfigurational transitions in certain transition‐metal systems (characterized by md^*n*^ electronic configurations) and lanthanides (with 4f^*n*^ configurations) [13, 14, 15]. Eu^3+^ ions were regarded as exemplary systems for the study of MD emission. A strong correlation was observed between the emission behavior and the point symmetry of the host lattice site. Incorporation of the ions into low‐symmetry noncentrosymmetric lattice sites resulted in the ED transition predominating the spectrum, which was noted for its acute sensitivity to the local chemical milieu of the luminescent center. The occupancy of centrosymmetric lattice sites by Eu^3+^ ions resulted in the MD transition becoming the dominant radiative pathway, which was characterized by its enhanced robustness against perturbations in the local crystal field [16, 17, 18, 19]. Therefore, the ratio of ED to MD emission intensity could be employed as a sensitive probe for tracking microenvironmental dynamics in the host material [14, 20, 21]. It was reported that the intensity of electromagnetic fields near metallic films or nanostructures could be quantified through luminescence measurements using Eu^3+^ ions. This approach exploited the distinct responses of ED and MD transitions to the local electromagnetic environment, enabling their relative intensities to serve as an intrinsic nanoscale probe [22, 23, 24].

The photonic environment emerged as a principal factor dictating the characteristics of light–matter interactions in lanthanide ions because of Purcell effect [25, 26]. A critical finding was that MD emitters shared a critical dependence on the photonic environment with ED emitters, extending beyond their intrinsic properties. This external influence was governed by distinct variants of the Purcell effect: the magnetic Purcell effect for MD transitions and its electric counterpart for ED transitions [10, 27, 28, 29]. A substantial research focus during the past decade was placed on the manipulation of spontaneous emission from both ED and MD transitions, achieved through the strategic implementation of resonant cavities [30, 31, 32, 33] and optical antennas [34, 35, 36, 37]. In the last few years, Montagnac et al. [38] achieved modulation of the spatial distribution of ED and MD emissions of Eu^3+^ ions using Si nanoantennas, through switching the laser polarization state. Bashiri et al. [39] achieved directional control of the fluorescence emission from Eu^3+^ complexes at MD (590 nm) and ED (610 nm) by employing a symmetry‐broken metasurface constructed from titanium dioxide nano‐resonators. Reynier et al. [40] demonstrated the selective excitation of Y_2_O_3_:Eu^3+^ nanoparticles (NPs) through the magnetic field of a localized plasmon. Achieved by employing a plasmonic nanoantenna., Gangrskaia et al. [41] reported a 3.0–4.5‐fold selective enhancement of the MD transition in Y_2_O_3_:Eu^3+^ thin file integrating in the cylindrical metallic antenna and exciting with an azimuthally polarized beam. The confinement of optical fields to deeply subwavelength volumes by plasmonic nanostructures was achieved, yielding a massive enhancement of the electromagnetic field intensity and the local density of optical states (LDOS). Subsequent studies established that metal‐based cavities and nanospheres (NSs) dimers provided a viable method for tuning the emission spectra of ED emitters through controlled alterations of the spectral profile of the LDOS [42, 43, 44]. Table 1 summarizes recent advances in the manipulation of the ED and MD emissions of Eu^3+^ ions. This control was primarily achieved by adjusting the distance of emitter‐mirror and the structural dimensions of the plasmonic nanocavity.

**TABLE 1:** Advances in modulating the fluorescence emissions of ED and MD in Eu^3+^ ions.

Configuration	Main transition	Method	Year	Reference
Eu(DBM)_3_phen	^5^D_0_→^7^D_1,2_	Control the emitter–mirror (Au) distance with spacer (SiO_2_)	2010	[17]
KY_7_F_22_: Eu^3+^	^5^D_0_→^7^D_1,2,4_	Control the emitter–mirror (Au) distance with SNOM tip	2014	[10]
KY_7_F_22_: Eu^3+^	^5^D_0_→^7^D_1,2,4_	Control the emitter position in plasmonic cavity (Al)	2018	[33]
Eu(DBM)_3_phen	^5^D_0_→^7^D_1,2_	Control radius of NPs (Si) coupled with emitter	2021	[20]
Y_2_O_3_:Eu^3+^	^5^D_0_→^7^D_1,2_	Control the emitter–mirror (Al) distance with SNOM tip	2023	[37]
CaF_2_:Eu^3+^	^5^D_0_→^7^D_1,2,4_	Control the shell (SiO_2_) thicknesses of plasmonic cavity (Ag) within emitter	2025	[42]

In this paper, the study presents a tunable plasmonic nanostructure composed of Au nanospheres (NSs) and an Au flake, designed to effectively modulate multiple ED and MD transition emission from Eu^3+^ ions doped NaYF_4_ NPs. The research demonstrates how distinct self‐interference effects between ED and MD transitions can be exploited for spectral modulation of NaYF_4_:Eu^3+^ emission. Atomic force microscopy (AFM) was employed as a nanomanipulation tool for precise positioning and assembly of nanoscale particles across diverse substrates [45, 46]. The emitters were positioned at controlled distances from steerable Au NSs to systematically modulate both electric and magnetic LDOS, enabling investigation of spontaneous emission rate variations in NaYF_4_:Eu^3+^ NPs. This competition between radiative decay channels (ED vs. MD) governed the relative emission intensities of transitions originating from identical excited states. Furthermore, the developed versatile platform permits scalable expansion to more complex nanostructures, where selective enhancement of either MD or ED transitions can be achieved through Purcell effect engineering. Through quantitative analysis of the relative intensities between spectrally adjacent transitions, the spatial distribution of optical modes within the system could be mapped. This approach offers a powerful means to nanoscale display, visualize nanoscale field distributions and their influence on quantum emitter behavior.

## 2 Results and Discussion

To demonstrate the self‐interference effect and the influence of plasmons on the ED and MD emissions of the NPs, a plasmonic nanostructure with controllable spacing was designed and fabricated on an atomically flat Au flake. The conceptual diagram of the entire experimental system is presented in Figure 1a. Figure 1b illustrates the mechanism of AFM‐based manipulation of Au NSs and control of interparticle distances as schematically depicted in the inset of Figure 1a. Experimental setup and sample preparation are elaborated on supporting information. In conventional AFM operation, dynamic scanning mode is typically employed, where the tip–sample distance is regulated by atomic force feedback to enable surface imaging. Upon deactivation of the force feedback, the AFM tip ceases to perform surface characterization and instead follows predefined trajectories. Precise adjustment of operational parameters and tip pathways allows controlled displacement of Au NSs or other nanostructures. The detailed manipulation procedures and parameters are provided in the Supporting Information S1 (Figure S1). The Au flake and NSs hybrid system were selected for its ability to support a resonance in the spectral range with both the electric and magnetic dipole transitions of Eu^3+^ ions.

**FIGURE 1:**
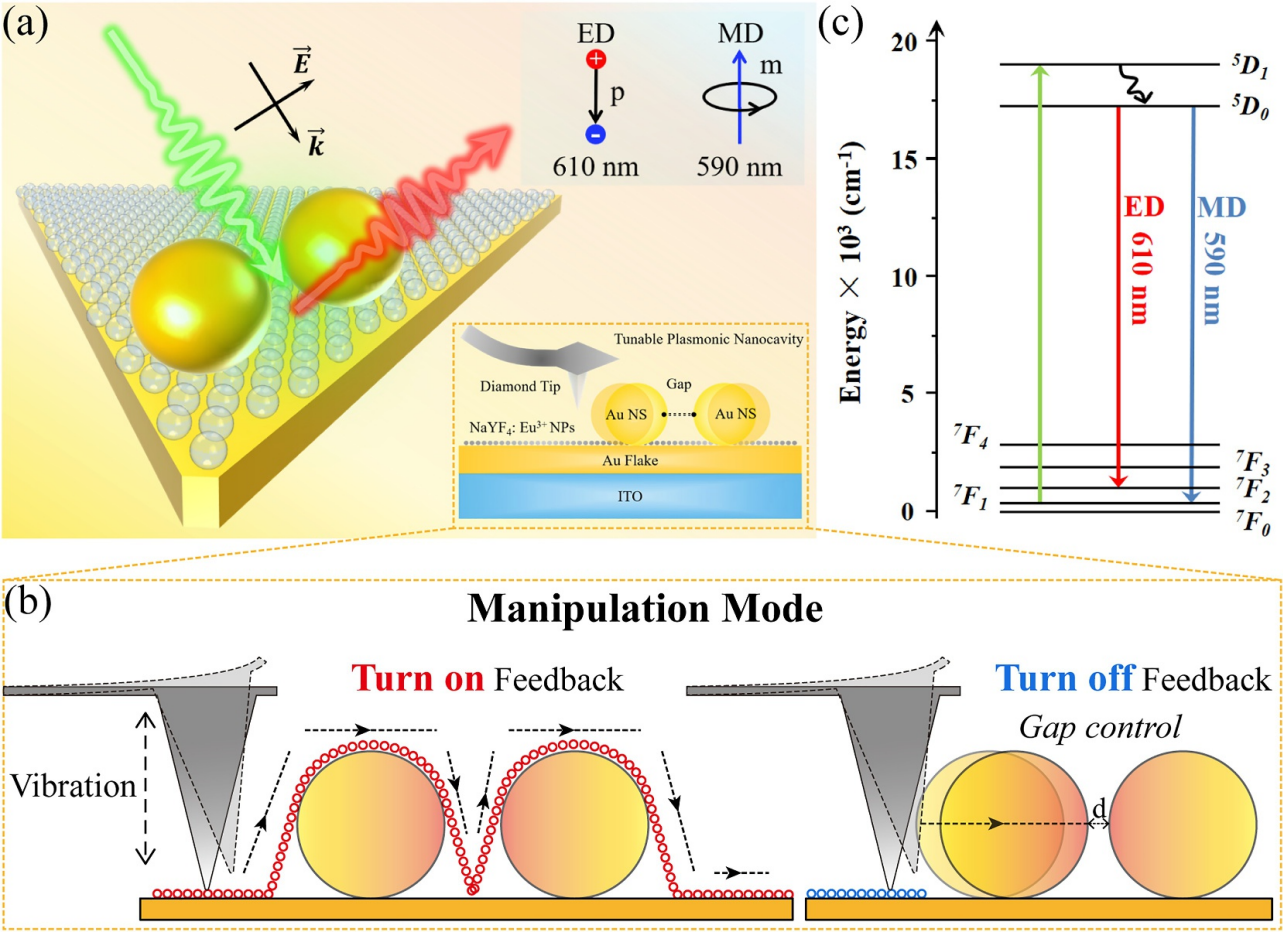
Illustration of the experimental setup and technical mechanism. (a) Schematic diagram of a gap‐controlled plasmonic nanostructure consisted of Au NSs and flake. Inset: the AFM with a diamond tip was used to manipulate the gap between to Au NSs. (b) Schematic of the mechanism by which AFM manipulates Au NSs. The switching between manipulation and characterization was achieved by toggling the force feedback of AFM on and off. The gap between Au NS dimmer was controlled through positional adjustment of the NSs after deactivation of the force feedback. (c) The energy level transition of Eu^3+^ ions doped in NaYF_4._

Figure 1c shows the energy‐level diagram of Eu^3+^ ions and the excited‐state decay pathways relevant to the experiments. A 532 nm excitation wavelength was selected, corresponding to the ED resonance transition from the ^7^F_1_ ground state to the ^5^D_1_ excited state (green arrow). The overall optical path was designed in an inverted excitation configuration (see Supporting Information S1, Figure S2). Following excitation, Eu^3+^ ions can decay down the ladder of energy levels by nonradiative relaxation pathways which was indicated with a black curve in Figure 1c. An exponential dependence on the phonon energy of the host material was observed for the rate of nonradiative multiphonon relaxation [1]. All these transitions were interconnected through the common ^5^D_0_ excited state. As a result, the suppression of one specific decay pathway was found to enhance the probability of radiative decay via the alternative channels [17].

A fundamental understanding of the physical principles regulating ED and MD transitions in Eu^3+^ ions has long been established as critically important. The initial observation of MD transition emission in optical systems was recorded approximately in 1940, through wide‐angle interference experiments employing Eu^3+^ ions [47]. A distinct environmental sensitivity was observed between transition types: the ED transition (^5^D_0_→^7^F_2_) demonstrated hypersensitivity to temperature and local coordination, whereas the MD transition (^5^D_0_→^7^F_1_) remained environmentally robust. ED and MD transitions represent distinct light–matter interaction mechanisms. These processes are mediated through oscillations of the ED moment for ED transitions and the MD moment for MD transitions, respectively [48].

The light–matter interaction between a charge‐neutral quantum system and an electromagnetic field was able to be described by a multipole expansion of the underlying interaction Hamiltonian [49].

(1) H=−p·E(t)−m·B(t)−[Q∇]·E(t)−⋯,

where ***p*** is the ED moment, ***m*** is the MD moment and ***Q*** is the electric quadrupole moment as a tensor. From a quantum electrodynamics perspective, ED and MD transitions represent distinct coupling pathways between electromagnetic fields and atomic systems—the former arising from charge displacement (***p·E*** coupling) and the latter from current loops (***m·B*** coupling). The different terms of the interaction Hamiltonian lead to different selection rules, and their magnitudes depend on the size of the quantum system. In an atomic system with the Bohr radius *a*_0_, the ED magnitude is given by *p = ea*_*0*_, where *e* is the elementary charge. The MD magnitude is given by the Bohr magneton *μ_B_ = ℏe/2m_e_*, where *m_e_* is the electron mass. In free space, the magnetic field of a plane wave satisfies *B = E/c*, where *c* is the speed of light. As a result, the MD interaction is weaker than its electric counterpart by a factor of *ℏ*/(*mca*_*0*_) = 1/137 = *α* [16]. From Figure 1c, it was observed that the common ED and MD transitions of Eu^3+^ ions were mutually coupled through the excited state ^5^D_0_. It followed that the inhibition of one transition resulted in an elevated probability of radiative decay through competing pathways. An ideal platform for studying this coupling is an emitter in proximity to a planar metal surface, a configuration in which the Purcell effect modifies the radiative decay rate of emitter. Physically, this modification is fundamentally a classical phenomenon arising from the self‐interference of the emitter with its own field reflected back to its origin.

A monolayer of NaYF_4_:Eu^3+^ NPs was uniformly deposited onto an Au flake to enable a systematic investigation of the self‐interference mediated modulation of the ED (^5^D_0_→^7^F_2_) and MD (^5^D_0_→^7^F_1_) transition intensities in Eu^3+^ as shown in Figure 2a. The supporting Au flake, synthesized through a wet‐chemical approach, was measured to have a thickness of 80 nm and demonstrated a surface roughness of approximately 0.5 nm, indicative of near‐atomic flatness as shown in Supporting Information S1: Figure S3. The monolayer of NaYF_4_:Eu^3+^ NPs deposited on the Au flake was synthesized through high‐temperature thermal decomposition, with an average particle size of 12 nm and a doping concentration of 20%. The dashed frame in Figure 2a indicates the region magnified in Figure 2d, where the uniformity of the monolayer is clearly delineated. A smooth monolayer film of NaYF_4_:Eu^3+^ NPs was achieved by spin‐coating, with the precise thickness being subsequently verified by AFM characterization as presented in Supporting Information S1: Figure S4. Furthermore, the crystalline structure of the NPs was confirmed by X‐ray diffraction (XRD) as shown in Supporting Information S1: Figure S5, with results consistent with the standard NaYF_4_:Eu^3+^ crystal structure.

**FIGURE 2:**
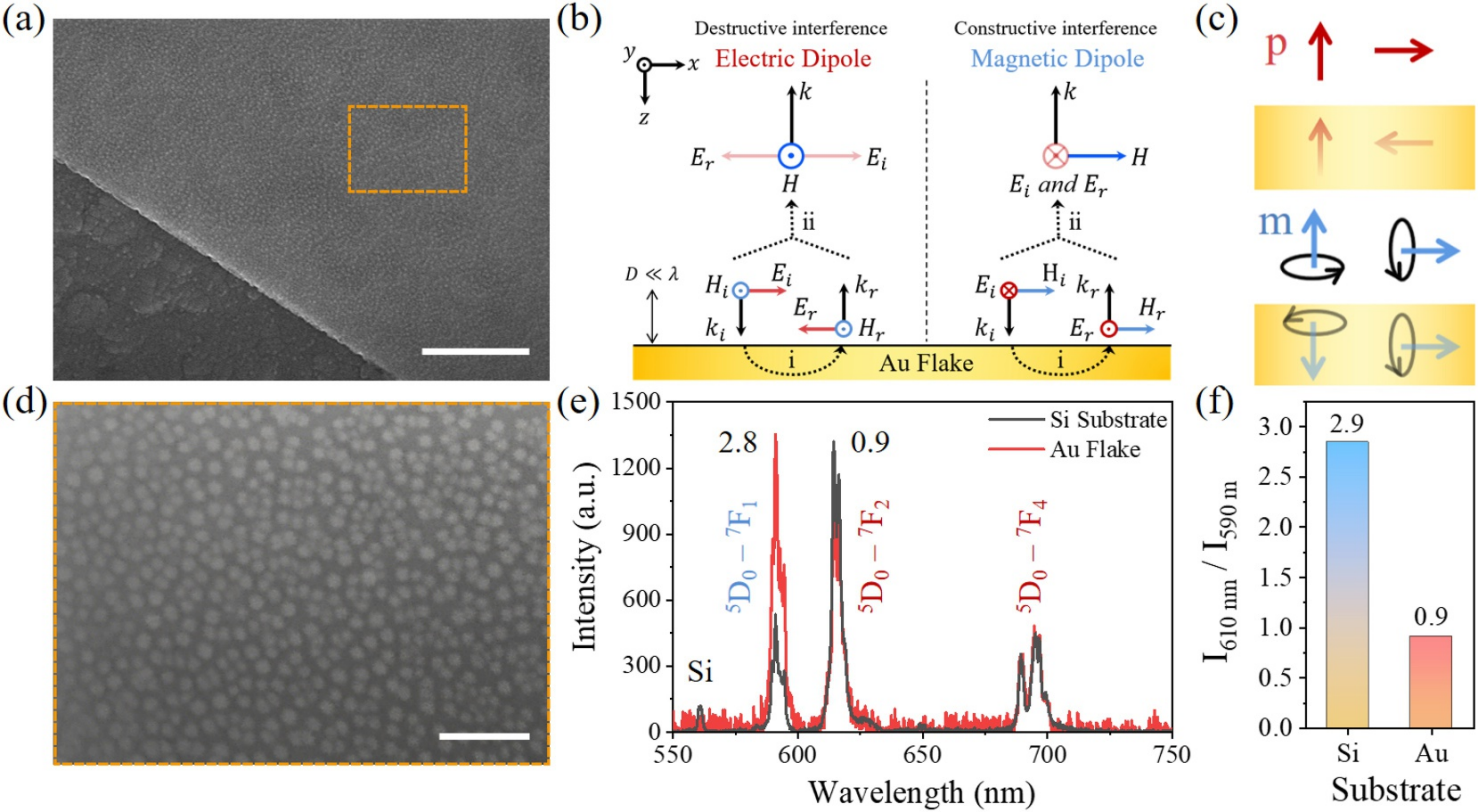
Fluorescence of NaYF_4_: Eu^3+^ NPs on different substrate. (a) Topographic image taken with an SEM showing an Au flake coating with a monolayer NaYF_4_: Eu^3+^ NPs. The scale bar is 500 nm. (b) schematic illustrating differing self‐interference effects for ED and MD emission along the *z* axis. The dashed lines indicate the light reflected by the Au flake interface. Owing to opposite field symmetries, MD emission into this mode is enhanced by constructive interference, whereas ED emission is suppressed by destructive interference. The underlying mechanism can be described by two distinct processes: (i) reflection and (ii) interference. (c) Dipoles near perfect mirrors: For electric dipoles perpendicular to the interface, interference is constructive; for dipoles parallel to the surface, interference is destructive; for magnetic dipoles, interference has the opposite effect. (d) The zoom‐in characterization of the monolayer NaYF_4_: Eu^3+^ NPs at the amber‐dashed outline as specifically indicated in (a). The scale bar is 200 nm. (e) The emission spectra of NaYF_4_:Eu^3+^ NPs on Si and Au flake substrate upon excitation at 532 nm with (f) a ratio of fluorescent luminescence intensity of the 610 nm peak to the 590 nm peak (I_610_/I_590_).

The distinct symmetry and polarization characteristics of ED and MD emissions were proved. Although both emissions were found to follow the sin^2^θ radiation pattern characteristic of point dipoles, their electric and magnetic field vectors were demonstrated to be interchanged [14]. The distinct field symmetries of ED and MD gave rise to complementary modifications, where the enhancement of one transition type typically occurred concurrently with the suppression of the other. Figure 2b shows that the electric field of an ED transition was symmetric under mirror reflection, in contrast to its magnetic field, which was antisymmetric, a fundamental difference that originated from their respective source characteristics [17]. A reciprocal symmetry relationship was observed for MD radiation, wherein the magnetic field maintained symmetry and the electric field exhibited antisymmetry under mirror transformation. Under normal incidence, a π‐phase shift, characteristic of a half‐wave loss, was introduced in the electric field component upon its reflection at the air–metal interface [17]. Given that the emission wavelength *λ* of the NaYF_4_:Eu^3+^ NP monolayer substantially exceeded its separation distance *D* from the Au flake ( *D* << *λ*), only single‐reflection events in the near‐interface region required consideration. Accordingly, as anticipated by the initial theoretical framework, the MD transition emission from Eu^3+^ ions was enhanced via constructive interference at the Au flake interface, whereas the ED transition emission was suppressed due to destructive interference. This interference behavior was further elucidated using the conceptual model of image dipoles, as schematically represented in Figure 2c [13]. According to the image dipole model, ED exhibited contrasting behaviors depending on their orientation relative to a mirror interface: parallel dipoles experienced suppression, while perpendicular dipoles showed enhancement in their transition probabilities. This trend was determined to be reversed for the case of a MD near the same boundary. Therefore, when spectroscopic measurements were conducted along the surface normal, an enhancement of MD transition intensity was observed alongside attenuation of ED transition intensity. This effect originated from the phase‐dependent interference between the direct emission and the field from its image dipole.

To verify the effect on the ED and MD transition emissions of Eu^3+^ ions, a 532 nm continuous‐wave laser was employed to excite a monolayer of NaYF_4_:Eu^3+^ NPs deposited on both Au flakes and Si substrates and the experimental results are presented in Figure 2e. A comparative analysis revealed that when NaYF_4_:Eu^3+^ NPs were placed on Au flake; significant modifications were observed in both the fluorescence peak intensities and the integrated intensity ratio between the ED (^5^D_0_→^7^F_2_) and MD (^5^D_0_→^7^F_1_) transitions compared to those on Si substrate. Specifically, the integrated emission intensity of the MD transition was enhanced by a factor of 2.8, whereas that of the ED transition was reduced to 0.9 times its original value. Since the excitation wavelength was 532 nm, the emission of Eu^3+^ ions was predominantly governed by the ED transition from the ^7^F_2_ state. Due to the self‐interference effect, not only was the ED emission suppressed near the Au flake but far‐field ED excitation was also inhibited. Additionally, the Au flake itself exhibited stronger background fluorescence. Consequently, as shown in Figure 2e, the emission spectrum of Eu^3+^ near the Au flake demonstrated significantly higher noise levels compared to that on the Si substrate. Although Eu^3+^ ions exhibit significant sensitivity to the local coordination environment, this behavior is exclusively observed in the hypersensitive electric dipole transition of ^5^D_0_→^7^F_2_. The intensities of other electric dipole transitions, such as ^5^D_0_→^7^F_4_, remain largely unchanged. This consistency was demonstrated by the minimal variations observed in the corresponding spectral emission profiles. Regarding self‐interference effects, both the nature of the dipole and the wavelength are influential factors. In Supporting Information S1: S6, self‐interference effects will be discussed in detail to explain the emission phenomena across different transitions. The spectral comparison confirmed two theoretical predictions: (1) Owing to the distinct field symmetries of ED and MD transitions, their decay rates exhibited a negative correlation. (2) Due to self‐interference effects, when compared to the reference case of a Si substrate without reflective metallic surfaces, the MD emission of Eu^3+^ ions on Au flake was significantly enhanced, whereas the ED emission was attenuated. As shown in Figure 2f, the integrated intensity of the ED transition at 610 nm (I_610_) from Eu^3+^ ions was normalized to the corresponding MD transition intensity (I_590_) for different substrates.

Previous studies have demonstrated the manipulation of ED and MD emissions from Eu^3+^ ions through self‐interference effects at Au‐flake‐based metallic mirror surfaces. In these systems, Eu^3+^ ions doped emitters exhibited strong intrinsic MD transitions that could dominate over ED transitions. The currently established model involves emitters near planar metal surfaces, where both ED and MD radiative decay rates are modified by the Purcell effect according to the emitter‐surface distance [1, 10, 37]. The LSPR effect of noble metals has been extensively utilized to modulate luminescence in Eu^3+^ ions doped NPs. Through modification of the local electromagnetic environment of emitters via LSPR, spontaneous emission rates can be effectively tuned, enabling precise luminescence control. To systematically investigate the combined effects of plasmonic interactions and mirror symmetry on ED and MD emissions, varying quantities of Au NSs were strategically deposited on Au flake surfaces. These Au NSs serve dual functions: not only forming nanocavities with the underlying flakes but also disrupting the intrinsic mirror symmetry of the planar Au surfaces. Moreover, through precise control of the interparticle spacing between Au NS dimers, dynamic tuning of the LSPR effects was achieved, enabling in situ modulation of the dipole emissions from Eu^3+^ ions.

The investigation of single Au NS effects on Eu^3+^ ion ED and MD emissions was conducted as shown in Figure 3. The dispersion density on Au flake surfaces was precisely regulated to guarantee the presence of only one Au NS within each excitation laser spot area. Sample morphology was characterized by AFM, as presented in Figure 3a. Individual Au NS positions were indicated by dashed frame, with NS diameters of approximately 60 nm being determined through line profile analysis along the marked dashed lines as shown in Figure 3c. Additional structural characterization was performed using SEM in Supporting Information S1: Figure S7, which further verified both the morphological uniformity of Au NSs and their optimal dispersion density on the Au flake substrates.

**FIGURE 3:**
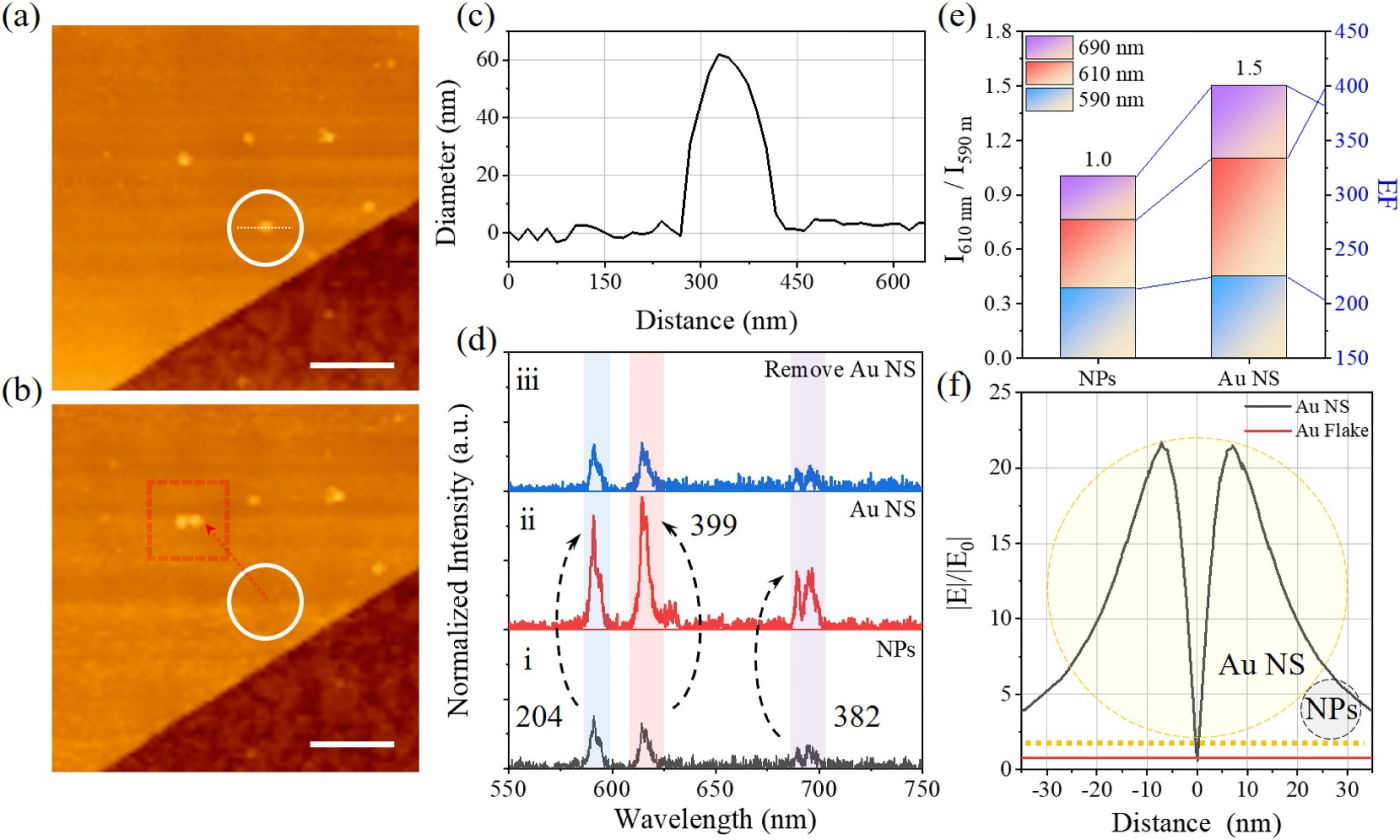
An impact of one single Au NS on emission of NaYF_4_: Eu^3+^. (a) AFM topographic image of one Au NS deposited on an Au flake which was located by a frame. The scale bar is 1 μm. (b) Marked Au NS was manipulated with AFM coupling to the second one. The motion path was indicated by red dashed line. The scale bar is 1 μm. (c) AFM profile of a 60 nm diameter Au NSs corresponding to the dashed line in (a). (d) The in situ emission spectra of NaYF_4_:Eu^3+^ NPs with (ii) or without (i) an Au NS on the Au flake upon excitation at 532 nm. All the spectral intensity was normalized to a reference value of (ii). The emission enhancement ratios at 590 nm (MD), 610 nm (ED), and 690 nm (ED) under the influence of a single Au NS were indicated by dashed lines, demonstrating enhancement factors of 204, 399, and 382, respectively. (e) A ratio of fluorescence intensity of the 610 nm peak to the 590 nm peak (I_610_/I_590_) with or without a single Au NS. The enhancement factors were more clearly demonstrated in panel (d). (f) The electric field enhancement results were presented along the yellow dashed line, with the red solid line and black contour representing the pristine Au flake surface and the system with deposited Au NSs, respectively.

The fluorescence spectrum of monolayer NaYF_4_:Eu^3+^ NPs on Au flake was established as the reference spectrum (Figure 3d‐i), exhibiting characteristic spectral features consistent with previous results in Figure 2 where the MD transition emission was weaker than the ED transition emission, yielding an integrated intensity ratio I_610_/I_590_ of 1.0. Upon excitation at the Au NS location marked by the dashed frame in Figure 3a, significant spectral modifications were observed in the corresponding spectrum (Figure 3d‐ii), including not only substantial emission enhancement but also a remarkable increase in the ED/MD transition intensity ratio to approximately 1.5. Detailed analysis revealed comprehensive emission enhancement across all energy levels, with the ED transition exhibiting significantly stronger enhancement compared to the MD transition. Since the fluorescence enhancement area of the nanocavity was small relative to the area of laser excitation, the average enhancement factor (EF) of the fluorescence emission intensity from the nanocavity coupled ED and MD transition emissions relative to those on the Si substrate was quantified according

(2) $$\text{EF}=\frac{I_{\text{NS}}-I_{\text{NPs}}}{I_{\text{NPs}}}\times \frac{N_{\text{spot}}}{N_{\text{NS}}},$$

where $I_{\text{NS}}$ is the specific emission (ED or MD) intensity from monolayer NaYF_4_:Eu^3+^ NPs with a single Au NS under an excitation spot with a diameter of 1 μm and $I_{\text{NP}}$ is the emission intensity from monolayer NaYF_4_:Eu^3+^ NPs without a single Au NS under the same laser excitation. The substrate in each condition is Au flakes. $N_{\text{spot}}$ is the number of the NPs within excitation laser spot about 3846, and $N_{\text{NS}}$ is the number of the NPs between the Au NS and Au flake about 18. It was found that the fluorescence emission intensity of MD (590 nm), ED (610 nm) and ED (590 nm) were enhanced by factors of 203.7, 399.0 and 381.6 relative to Au flake (without Au NS). These spectral modifications, including both the I_610_/I_590_ ratio variation and corresponding energy‐level specific changes, were systematically compared and presented in Figure 3e, demonstrating the pronounced effects of localized surface plasmonic enhancement on the dipole emission characteristics of Eu^3+^ ions.

The observed spectral differences between Figure 3d‐ii and 3d‐i were attributed to the introduction of a single Au NS, suggesting that the local spectrum should match the reference spectrum upon Au NS removal. This hypothesis was verified through precise nanomanipulation using AFM where the Au NS in the dashed square of Figure 3a was relocated along the red dashed arrow as shown in Figure 3b. Subsequent in situ spectral measurements at the original position after NP removal revealed nearly identical spectra to the reference as demonstrated in Figure 3d‐iii. The minor variation in 610 nm (ED) fluorescence emission was attributed to the disruption of symmetry in the monolayer Eu^3+^ ions doped NP array caused by the removal of Au NS, which consequently weakened the suppression of ED transition emissions through self‐interference effects. Controlling experiments conducted on Si substrates under identical conditions showed no spectral variations regardless of Au NS presence (Supporting Information S1: Figure S8), confirming that the plasmonic effects originated specifically from the Au NS‐Au flake hybrid system. Furthermore, finite element method (FEM) simulations comparing the electromagnetic field distribution of a single Au NS on a Si substrate with that of an isolated Au NS reveal that, unlike the Au flake, the Si substrate does not effectively modify the local electromagnetic environment as demonstrated in Supporting Information S1: Figure S9. These results demonstrate that a single Au NS on Au flake can generate nanocavity‐induced LSPR effects along its periphery, leading to overall fluorescence enhancement of Eu^3+^ ions. The FEM simulations presented in Figure 3f revealed the electric field enhancement factors beneath individual Au NS. Additional electromagnetic field distribution simulations for a single Au NS on an Au flake at the excitation wavelength (532 nm) and emission wavelengths (590, 610, and 690 nm) were performed. The results clarify that the fluorescence enhancement of Eu^3+^ ions by a single Au NS primarily originates from an excitation enhancement mechanism. Detailed results are presented in Supporting Information S1: Figure S10. The collective results were found to conclusively validate the initial theoretical analysis.

In addition to the LSPR‐mediated fluorescence enhancement of Eu^3+^ ions, the modified emission characteristics should also be attributed to the symmetry‐breaking‐induced attenuation of self‐interference effects near the Au flake surface. The introduction of Au NSs disrupts the mirror symmetry of the Au flake, leading to significant modifications in both MD and ED transition emissions of Eu^3+^ ions. This symmetry‐breaking effect results in weakened self‐interference at the metal‐dielectric interface, which consequently alters the relative emission intensities between MD and ED transitions. The weakening of self‐interference effects can also be verified by depositing non‐uniform multilayers of NaYF_4_:Eu^3+^ NPs on the Au flake surface, as shown in Supporting Information S1: Figure S11.

The nanomanipulation capability of AFM was utilized to precisely control the interparticle spacing between Au NS dimers, enabling investigation of the localized surface plasmonic effects on both electric dipole and magnetic dipole transitions of Eu^3+^ ions. Figure 4a shows the single Au NS corresponding to the square region in Figure 3a, whereas Figure 4d presents the normalized dark‐field scattering spectrum (indicated by black dots) and fluorescence emission spectrum obtained from the single NS. The dark‐field scattering spectrum of the single Au NS was observed to exhibit poor spectral overlap with both the electric dipole and magnetic dipole emission bands of Eu^3+^ ions. This is attributed to the misalignment between the scattering resonance of the small Au NS and the emission peaks of the ED and MD transitions. In Supporting Information S1: Figure S12, the simulated scattering spectrum of a single Au NS on an Au flake is presented. The nanocavity formed by a single Au NS and an Au flake exhibited a fixed mode volume, preventing dynamic tuning of the electric field distribution to further manipulate the dipole transitions of Eu^3+^ ions. In contrast, the nanocavity composed of a tunable Au NS dimer and an Au flake allowed precise control over the plasmonic interactions.

**FIGURE 4:**
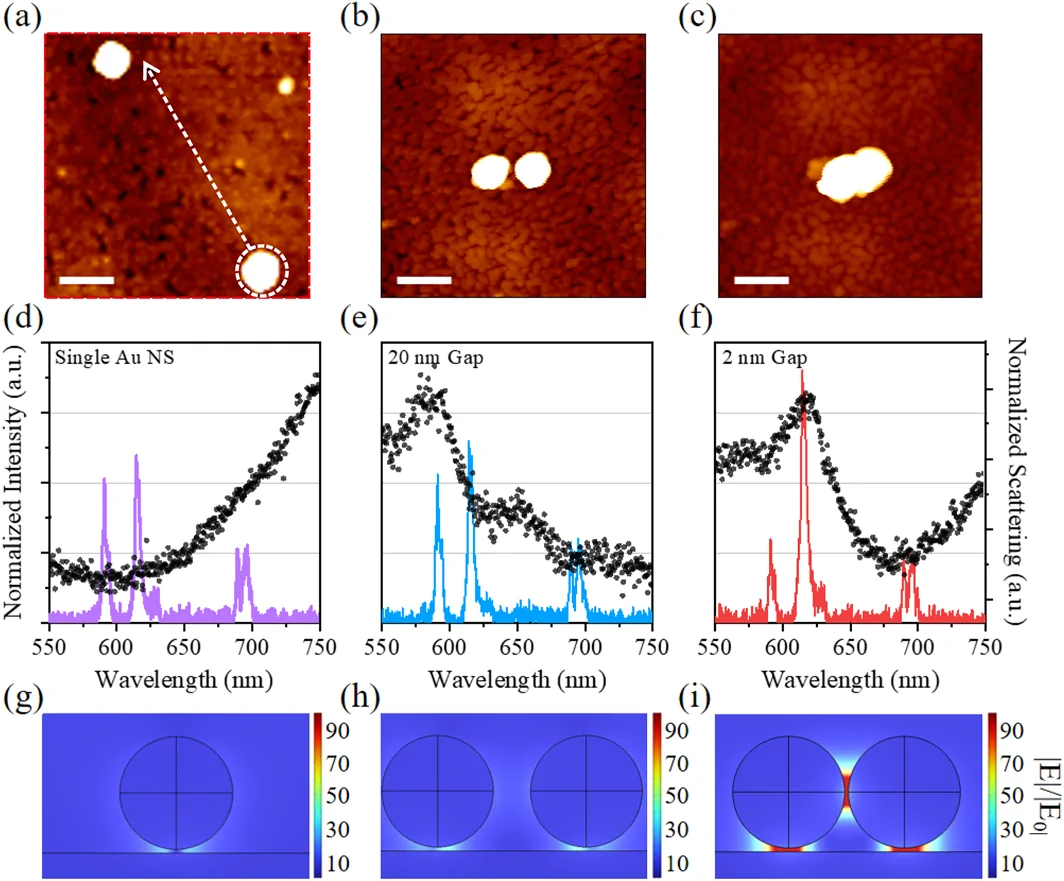
Gap manipulation of double Au NSs and the regulation of nano‐structured optical properties. (a) AFM topography image of two single Au NSs before manipulated. The Au NS position and movement path was exhibited with dashed line. The overall AFM characterization corresponds to the red dashed frame region in Figure 3b. (b) and (c) AFM topography image of double Au NSs with 20 and 2 nm gap, respectively. The scale bar is 200 nm. (d) Experimental dark‐field scattering (black dot) and fluorescence spectra (color) of the single Au NS. The same acquisition was at different interparticle distances of (e) 20 nm and (f) 2 nm. The electric field distributions in the *x*–*z* plane were simulated under 532 nm excitation for three configurations: (g) a single Au NS, (h) an Au NS dimer with 20 nm spacing, and (i) an Au NS dimer with 2 nm spacing. The maximum electric field enhancement factors are 23.2, 28.9, and 88.6, respectively.

The AFM characterization of Au NS dimers with precisely controlled spacings of 20 and 2 nm is presented in Figure 4b,c respectively. It should be noted that the resolution of AFM measurements is fundamentally limited by the tip curvature radius, unlike conventional characterization techniques. Complementary SEM imaging provided in Supporting Information S1: Figure S13 was utilized to confirm the actual interparticle spacing when the AFM resolution proved insufficient for accurate NS morphology determination. To prevent electron beam‐induced alterations to both the Au NSs and Eu^3+^ ions doped NPs during SEM characterization, in situ SEM analysis was exclusively performed for the 2 nm interparticle spacing configuration. For the dimer configuration with 20 nm spacing shown in Figure 4b, the corresponding dark‐field scattering spectrum in Figure 4e displayed a dominant resonance peak at 590 nm, which spectrally aligned with the ^5^D_0_→^7^F_1_ MD transition of Eu^3+^ ions. This observation was further supported by numerical simulations of the electric field distributions with FEM, where the results shown in Figure 4g for single NS and Figure 4h for 20 nm‐spaced dimer revealed minimal variations, indicating insufficient field enhancement at this spacing to significantly modify the optical LDOS. When the interparticle spacing was reduced to 2 nm as shown in Figure 4c, a distinct red‐shift of the scattering peak to 610 nm was observed in the corresponding spectrum shown in Figure 4f, now coinciding with the ^5^D_0_→^7^F_2_ ED transition. Quantitative analysis revealed that compared to the Au NS‐free case, this 2 nm‐spaced dimer configuration produced a 481.1‐fold enhancement in ED emission and a 16.1‐fold enhancement in MD emission while simultaneously raising the intensity ratios (I_610_/I_590_) to 4.3. All spectral data were normalized to enable direct comparison. The specific results regarding MD (590 nm) and ED (610 nm) transition emission EFs, along with ED/MD emission intensity ratios, were presented in Table 2 for monolayer NaYF_4_:Eu^3+^ NPs under three distinct conditions: on Au flakes, under the influence of a single Au NS and with two Au NSs at varying separation distances, all compared against the reference case without Au NS.

**TABLE 2:** The emission characteristics of distinct transitions in monolayer NaYF_4_:Eu^3+^ NPs with and without the influence of Au NSs.

Au NS condition	EF of MD (590 nm)	EF of ED (610 nm)	I_610 nm_/I_590 nm_
No Au NS	\	\	0.99
One Au NS	203.7	399.0	1.46
Two Au NSs (20 nm gap)	110.8	229.7	1.54
Two Au NSs (2 nm gap)	16.1	481.1	4.30

To further elucidate the photon dipole transition radiation process of Eu^3+^ ions under localized surface plasmon regulation, the transition branching ratio was introduced to investigate the redistribution of emission channels influenced by the photonic LDOS, which was expressed by the following formula [11, 35]:

(3) $$\beta_{i}=\frac{I_{i}}{I_{\text{tot}}}=\frac{\Gamma_{i}^{\text{rad}}}{\Gamma_{\text{tot}}^{\text{rad}}},$$

where $I_{i}$ is the integrated intensity of the transition labeled *i* (MD or ED), $I_{\text{tot}}$ is the sum of the three contributions, $\Gamma_{i}^{\text{rad}}$ is the radiative emission rate of the transition labeled *i* and $\Gamma_{\text{tot}}^{\text{rad}}$ is the sum of the radiative emission rates of each transition. Thus, the relative LDOS experienced by the ED and MD transitions could be quantitatively determined as follows [11, 35]:

(4) $$\rho_{\text{ED},\text{MD}}=\frac{\beta_{\text{ED},\text{MD}}\cdot f_{\text{MD},\text{ED}}}{\beta_{\text{ED},\text{MD}}\cdot f_{\text{MD},\text{ED}}+\beta_{\text{MD},\text{ED}}\cdot f_{\text{ED},\text{MD}}},$$

where $\rho_{\text{ED},\text{MD}}$ are the electric or magnetic relative radiative LDOS and $f_{\text{ED},\text{MD}}$ are the oscillator strengths for the electric or magnetic transitions, respectively. The value of the branching ratio $\beta_{\text{ED},\text{MD}}$ measured at large distance to the Au flake was used as an estimate of $f_{\text{ED},\text{MD}}$. This method enabled the correlation between spectrally resolved emission intensities in steady‐state photoluminescence experiments and the electric and magnetic LDOS. In Supporting Information S1: Figure S14a, the energy levels of Eu^3+^ ions were simplified to a four‐level system for theoretical analysis. Supporting Information S1: Figure S14b presents the variation in branching ratios between the ^5^D_0_→^7^F_2_ ED transition and the ^5^D_0_→^7^F_1_ MD transition as a function of decreasing interparticle spacing in Au NS dimers, with experimental data derived from Figure 4. A linear correlation was observed between the interparticle distance and the transition branching ratios—the ED transition emission was enhanced whereas the MD transition emission was suppressed with reduced spacing. This inverse relationship demonstrates parallel evolution in the radiative decay rates of both transition types under plasmonic modulation. Consequently, by monitoring the fluorescence emission, particularly the ratio between ED and MD transition emissions at varying interparticle distances of Au NS dimers, the distribution of electric and magnetic LDOS in plasmonic nanostructures could be inferred with nanoscale precision.

As the gap distance between the Au NS dimers decreased, the variation in ED and MD transition emissions of Eu^3+^ ions could be attributed to two factors. First, the reduced gap provided stronger localized field enhancement, which increased the spontaneous emission rate of the ED transition, thereby enhancing its emission. Second, at a 2 nm gap distance, the resonance enhancement of the entire nanostructure precisely matched the ED transition emission peak, further amplifying its emission. In Supporting Information S1: Figure S15, the magnetic field distributions for a single Au NS, a 20 nm, and a 2 nm gap Au NS dimer are provided, demonstrating the complementary variations of the electric and magnetic fields and their impact on the ED and MD transition emissions of Eu^3+^ ions. As previously discussed, the emission characteristics of Eu^3+^ ions’ ED and MD transitions were interdependent—enhancement of ED emission inevitably led to suppression of MD emission. As illustrated in Figure 4i, strongly coupled Au NS dimers at this separation induced significantly intensified LSPR effects. At this gap distance, electron tunneling has not yet initiated, allowing the LSPR effect to provide stable electric field enhancement [50]. Consequently, the influence of LSPR on ED transition emissions of Eu^3+^ ions reached its maximum magnitude, thereby suppressing MD transition emissions. When the Au flake was replaced with a Si substrate while keeping other conditions unchanged, the fluorescence spectrum of Eu^3+^ ions exhibited negligible changes as shown in Supporting Information S1: Figure S16. The minimal alterations observed in Eu^3+^ ions emission characteristics—both for single Au NS and dimers with separations exceeding 20 nm—further confirmed that only strongly enhanced and spectrally matched LSPR effects could effectively modulate and enhance these transitions.

The preceding analysis demonstrated that the emission capabilities of Eu^3+^ ions were directly correlated with the electric and magnetic photonic quantum environment of the nanocavity. Consequently, the number of Au NSs on the Au flake could be also precisely controlled to manipulate the near‐field electric or magnetic LDOS, thereby influencing and regulating the emission properties of Eu^3+^ ions. Figure 5a illustrates the AFM‐controlled assembly process of Au NSs, accompanied by corresponding AFM characterization. Supporting Information S1: Figure S17 presents the topographic profiles at different stages and ultimate status, clearly delineating the actual contours of the NSs. Initially, only a single Au NS was positioned within the excitation spot, and the emission spectrum of the monolayer NaYF_4_:Eu^3+^ NPs was recorded as shown in Supporting Information S1: Figure S18.

**FIGURE 5:**
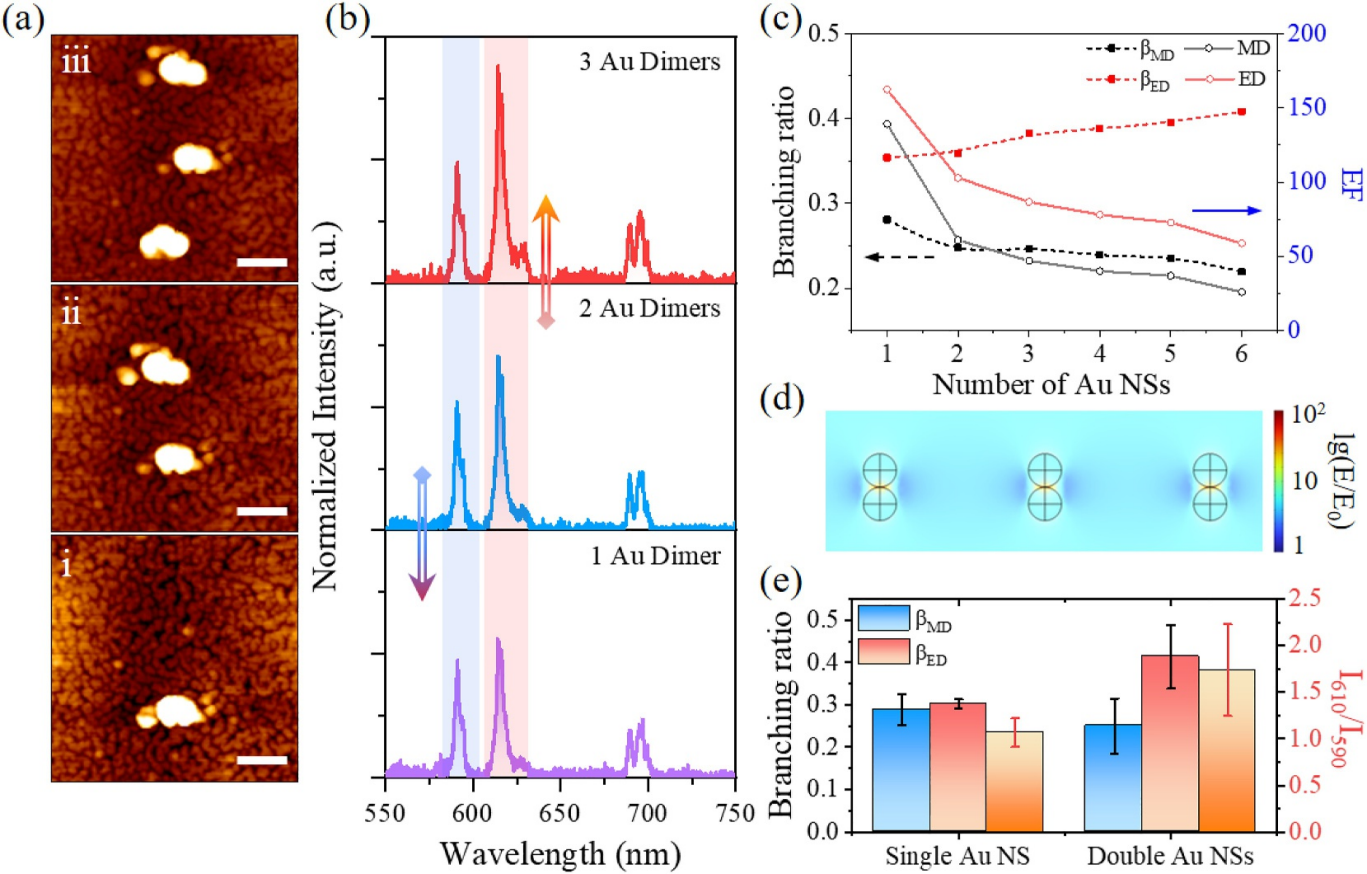
Modulating the fluorescence emission properties through precise control of Au NS quantities. (a) AFM topographic image of Au NSs varying quantities. (i), (ii), and (iii) corresponded to a single pair, two pairs, and three pairs of Au NSs, respectively. AFM‐based nanomanipulation was employed to construct all pairs of Au NSs, with a fixed separation distance of 2 nm between adjacent particles. The scale bar is 200 nm. (b) The effect of varying pairs of Au NSs on the fluorescence emission of monolayer NaYF_4_:Eu^3+^ NPs. As the number of Au NSs dimers increasing, the ED emission of Eu^3+^ ions was gradually enhanced, whereas the MD emission was weakened significantly. (c) Under varying numbers of Au NSs, the enhancement factors (solid lines) and branching ratios (dashed lines) were systematically characterized for both the MD and ED transitions of Eu^3+^ ions. (d) The electric field enhancement distribution was characterized for six Au NSs positioned on an Au flake. (e) The branching ratios of MD transitions, ED transitions, and the ED/MD intensity ratios were systematically investigated for monolayer NaYF_4_:Eu^3+^ NPs under the influence of both individual Au NS and Au NS dimers.

Subsequently, AFM manipulation was utilized to construct Au NS dimers. The assembly process, spanning from Figure 5a‐i–iii, resulted in three pairs (six NSs in total) positioned within the excitation spot, with an interparticle spacing of approximately 300 nm for each pair. Since the construction of multiple dimers relied on sequential NS manipulation, the fluorescence spectra of the monolayer NaYF_4_: Eu^3+^ NPs were acquired after each dimers relocated. As depicted in Figure 5b, the increase in NS count reproduced the previously analyzed trend that the ED transition emission enhanced along with MD transition emission suppressed. Figure 5c quantifies the branching ratios and enhancement factors of ED and MD transitions under varying numbers of Au NSs. The quantities of Au NSs was progressively increased during the construction of coupled NS pairs, with laser spot being ensured to contain a corresponding quantity of NSs during excitation of the monolayer NaYF_4_:Eu^3+^ NPs. The emission spectra of Eu^3+^ ions doped NPs were acquired with varying numbers of Au NSs as presented in Supporting Information S1: Figure S18.

The LSPR effect between the Au NSs and the flake altered the surrounding electromagnetic environment, modifying the spontaneous emission rate of the Eu^3+^ ions doped NPs and manifesting as spectral intensity variations. Strengthening the plasmonic nanocavity structure was predicted to amplify this effect. Meanwhile, the observed variation in Eu^3+^ ions luminescence emission further indicated that weak interactions existed between each pair of Au NSs, resulting in gradual enhancement of the corresponding emission ratio with increasing numbers of dimers. To validate these, numerical simulations were performed to analyze the electric field distribution and enhancement around nanostructures with different NS counts as shown in Figure 5d. As anticipated, the field enhancement intensified with increasing NS density within the laser‐excited region. These results further corroborated the regulatory role of LSPR in the spontaneous emission of Eu^3+^ dipole transitions and highlighted that even localized electromagnetic field enhancement could exert a pronounced influence on the overall emission profile. Statistical analysis of multiple modulation experiments performed on both individual Au NS and Au NS dimers was presented in Figure 5e. The Au NS dimers were demonstrated to exhibit superior capability in modulating the branching ratio between the MD and ED transitions of Eu^3+^ ions compared to single NS. Upon interaction with Au NS dimers, a distinct reduction in the *β*_MD_ parameter was observed for monolayer NaYF_4_: Eu^3+^ NPs, whereas the *β*_ED_ parameter was found to be significantly enhanced. Furthermore, a remarkable increase in the integrated intensity ratio of ED to MD transitions (I_610_/I_590_) for Eu^3+^ ions was consistently recorded. By modulating the electromagnetic properties of the excitation environment through variations in nanostructure density, the spatial distributions of both electric LDOS and magnetic LDOS were successfully mapped based on the relative intensity variations between ED and MD emissions of Eu^3+^ ions. This methodology was demonstrated to hold significant implications for nanoscale electromagnetic field manipulation.

## 3 Conclusion

In conclusion, this study demonstrated a plasmonic nanostructure platform for precisely controlling ED and MD transitions in NaYF_4_:Eu^3+^ NPs through precisely engineered local photonic environments. By employing AFM nanomanipulation to construct tunable nanostructures composed of Au NSs and an Au flake, selective modulation of electric and magnetic LDOS was achieved, enabling independent control over ED and MD transition emissions from Eu^3+^ ions. This platform permitted emitter positioning with nanometer precision, facilitating systematic investigation of self‐interference effects on competing radiative pathways and their influence on ED/MD transition characteristics of Eu^3+^ ions. The spectral analysis of ED/MD transitions served as a sensitive probe for mapping nanoscale electromagnetic field distributions. Secondly, by leveraging the broken mirror symmetry and overlapping coupling with LSPR at the Au flake‐NP interface, the ratio between ED and MD transition emissions from Eu^3+^ ions was dynamically tuned from 1.0 to 4.3. Meanwhile, a remarkable enhancement of ED transition emission by a factor of 481 was simultaneously achieved with an accurate nanoscale gap regulation by this platform. The introduction of transition branching ratios enabled detailed examination of electromagnetic LDOS distribution in the near‐field regime and the Purcell effect’s role in enhancing the spontaneous emission rate of Eu^3+^ ions. Experimental results reproduced previously demonstrated emission modification outcomes achieved through conventional spacer‐layer strategies. Combined with theoretical simulations, these findings further verified that localized electric field enhancement could quantitatively regulate LDOS and radiative decay rate via branching ratios. The method offers directness, rapid implementation, and compatibility with diverse nanostructure geometries. This approach provides a versatile tool for fundamental studies of light–matter interactions and the development of advanced nanophotonic devices, with potential applications in quantum light sources, nano‐sensors, and other systems requiring controlled electric and magnetic transition components. Furthermore, its in situ spectral monitoring capability during operation establishes a robust framework for designing functional materials with tailored emission properties through controlled plasmonic electromagnetic environments.

## Author Contributions

**Chengxiang Gou:** conceptualization, methodology, investigation, formal analysis, writing – original draft, writing – review and editing, resources, visualization. **Bowen Kang:** software. **Wangze Li:** resources. **Xiangtai Xi:** resources. **Yuting Luo:** resources. **Jiaqi Wang:** resources. **Zhengkun Fu:** formal analysis, funding acquisition. **Huan Chen:** formal analysis, funding acquisition. **Zhenglong Zhang:** supervision, formal analysis, writing – review and editing, funding acquisition. **Hairong Zheng:** supervision, formal analysis, writing – review and editing, funding acquisition.

## Funding

This work was supported by the National Natural Science Foundation of China (Grants 12474389, U22A6005, 12574442, and 12304426), the National Key R&D Program of China (Grant 2024YFA1409900), the Natural Science Foundation of Shaanxi Province (Grant 2024JC‐ICON‐07), and the Fundamental Science Foundation of Shaanxi (Grants 23JSQ007 and 24JP040).

## Ethics Statement

The conducted research is not related to either human or animals use.

## Consent

Informed consent was obtained from all individuals included in this study.

## Conflicts of Interest

The authors declare no conflicts of interest.

## Supporting information


Supporting Information S1

## Data Availability

The datasets generated and/or analyzed during the current study are available from the corresponding author upon reasonable request.
