# Supplementary material for: Self‐Interfering Magnetic Dipole Transition Emission in Rare‐Earth Ions Doped Nanoparticles Modulated by Plasmonic Mirror Nanocavity
Source: Nanophotonics. 2026 Apr 10;15(7):e70065. doi: 10.1002/nap2.70065 (PMC13642099; doi:10.1002/nap2.70065)
Supplement: Supplementary file 1 [file NAP2-15-e70065-s001.docx]

Supporting Information

Self-Interfering Magnetic Dipole Transition Emission in Rare-Earth Ions Doped Nanoparticles Modulated by Plasmonic Mirror Nanocavity

Chengxiang Gou, Bowen Kang, Wangze Li, Xiangtai Xi, Yuting Luo, Jiaqi Wang, Zhengkun Fu, Huan Chen, Zhenglong Zhang* and Hairong Zheng*

School of Physics and Information Technology, Shaanxi Normal University, Xi’an

710062, China

*Corresponding authors:

Zhenglong Zhang- Email: zlzhang@snnu.edu.cn.

Hairong Zheng- Email: hrzheng@snnu.edu.cn.

**Experimental methods**

Experimental setup

The instruments used include: atomic force microscope (AFM) from Nanowizard Ultraspeed JPK, Berlin, Germany, Renishaw DPSS laser with 50 mW at 532nm, Olympus ix73 inverted microscope, 100x M Plan S- Apochromat objective lens with N.A. 0.9 and W.D.1.0 mm and High-resolution micro confocal Raman spectrometer from Renishaw inVia Spectrometer. The power density is 1.53×10^6^ W·cm^-2^. The sample were excited by observing position through the camera. The fluorescence spectrum was collected through the objective lens. Before being reflected to the spectrometer, the excitation light signal was filtered out through the filter.

Synthesis of NaYF4: Eu^3+^ nanoparticles (NPs).^[1]^

NaYF_4_: Eu^3+^ NPs were synthesized according to the thermal decomposition method. In short, 1.6 mmol of precursors Y(CH_3_COO)_3_, and 0.4 mmol Eu(CH_3_COO)_3_ (20% doped) were mixed with 12 mL of oleic acid and 30 mL of octadecene. The solution was heated to 160 ℃ under vigorous stirring to form a homogeneous solution and then cooled to room temperature. 10 mL methanol solution of NaOH (5 mmol) and 20 mL methanol solution of NH_4_F (8 mmol) were slowly added into the flask and stirred for 30 min. The solution was then slowly heated to remove methanol, degassed at 100 ℃ for 20 min, followed by heating at 305 ℃ for 25 min under argon protection. The NPs of NaYF_4_: Eu^3+^ were precipitated from the solution with ethanol and washed with ethanol and cyclohexane three times.

Synthesis of Au nanospheres (NSs).^[2]^

The Au NSs were obtained by a seed-mediated growth method with three steps. Briefly, a HAuCl4 solution (0.01 mol, 0.25 mL) was first mixed with a CTAB solution (0.1 mol, 9.75 mL), followed by the rapid injection of a freshly-prepared, ice-cold NaBH4 solution (0.01 mol, 0.60 mL) under vigorous stirring. The resultant solution was kept under gentle stirring for 3 h at room temperature. 0.12 mL of the as-prepared seed solution was injected into a growth solution made of CTAB (0.1 mol, 9.75 mL), water (190 mL), HAuCl4 (0.01 mol, 4 mL), and ascorbic acid (0.1 mol, 15 mL). The reaction mixture was gently shaken and then left undisturbed overnight at room temperature. The resultant small Au NS sample was washed and concentrated by four times into water by centrifugation and redispersion for further use. The Au nanopolyhedrons were grown by the seed-mediated method using the small Au NSs as seeds. Then, the seed solution 1 mL was first added into a CTAC solution (0.025 M, 30 mL). After the sequential addition of ascorbic acid (0.1 M, 0.75 mL) and HAuCl4 (0.01 M, 1.5 mL), the mixture solution was placed in an air-bath shaker (45 °C, 160 revolutions per minute) and kept for 3 h. The obtained Au nanopolyhedrons were centrifuged and redispersed in a CTAB solution (0.02 M, 30 mL). At length, the Au nanopolyhedrons in CTAB solutions were mixed with a HAuCl4 solution (0.01 M, 0.2 mL). The resultant mixture solution was kept in the airbath shaker (45 °C, 160 revolutions per minute) for 2 h. Au NSs were produced through the mild oxidation of the Au nanopolyhedrons in the presence of HAuCl4 and CTAB. The obtained Au NSs were centrifuged and redispersed in water for storage.

Synthesis of Au flake.^[3]^

Au flakes were synthesized by a wet chemical method. Gels of HAuCl_4_ solution (1.5 ml, [HAuCl_4_ - PVP (with molar ratios PVP/Au = 200–300) were obtained at 50°C by mixing HAuCl_4_] = 0.04 m) with PVP (K30). The gels were kept at 50°C for a 24 h period. Then the gels were transferred to an environment with temperature lower than 5°C. Finally, the single-crystal Au flakes were obtained by placing the gels at temperatures less than 50°C over a long period of time.

Assembly of monolayer NPs.

A two-step spin coating method was used to prepare a monolayer NaYF_4_: Eu^3+^ film. Primarily, the concentrated NPs stock solution was diluted thirty times in cyclohexane. The first step spin coating parameters were set to 250 rpm for 10 seconds and the second parameters were set to 500 rpm for 50 seconds. Launching the program, the 50 μL of disperse solution was transferred slowly with a drip on the substrate. Subsequently, the substrate was rinsed with pure cyclohexane and dried with nitrogen.

Fabrication of plasmonic nanostructures.

The plasmonic nanostructures was constructed on the Au flake by Au NSs, which was manipulated with nanometer accuracy using the tip of an AFM. The diamond tip of the AFM was used to position Au NSs mechanically on the Au flake. Then the Au NSs were assembled on the Au flake and the gap distance between the NSs was subsequently varied. This allows for varying and controlling the distance between the NSs with nm accuracy, which is 1 order of magnitude more precise compared to typical precisions achieved in electron beam lithography.

The finite element simulation.

All electromagnetic field distributions are calculated and simulated using the finite element method (FEM). The finite-element simulation software COMSOL Multiphysics 6.1 was used to model all structures. Given the size of the Au NSs and the focusing conditions (slightly defocused from the focal plane to ensure laser spot uniformity and balance effects from minor positional errors of the Au NSs), plane wave incidence was employed in the simulations.

S1. Performing nanomanipulation on Au NSs.

Figure S1. The AFM nanomanipulation process of Au NSs on a Si substrate was systematically investigated, as demonstrated in Figures (i)-(viii). The initial and final morphological configurations of the target Au NSs were characterized by scanning electron microscope (SEM) imaging in panels (i) and (viii) respectively, with regions of interest being demarcated by dashed frame. The scale bar is 1 μm. Precise spatial control at the nanoscale was achieved through optimization of AFM operational parameters, wherein the integral gain (I gain) and set point value were identified as the dominant control variables, being set at 10 Hz and 0.01 respectively, while the proportional gain (P gain) was found to exert negligible influence. The nanomanipulation procedure was conducted with incremental displacement steps ranging from 50 to 100 nm, at a constant scanning velocity of 0.05 μm·s^-1^. The controlled translocation trajectory was monitored through AFM topographical imaging in panels (ii)-(vii), through which the progressive movement of individual NS was clearly resolved. The scale bar is 200 nm. Parameter optimization was required when different substrate materials were employed.

S2. Schematic of experimental configuration.

Figure S2. The schematic illustrates the specific optical configuration employed in the experimental setup. The inverted microscopic optical system was utilized, with the sample mounted in an inverted orientation on the sample stage. A 532 nm linearly polarized laser beam, after beam expansion, was focused onto the sample through a 100× objective lens (NA 0.9). The sample stage, integrated with a precision translation mechanism, enabled accurate positioning with a minimum displacement resolution of 1 nm.

S3. Detailed characterization of Au flakes.

Figure S3. The Au flakes were characterized using AFM (a) and SEM (b), with a scale bar of 5 μm. (c) The height profile along the dashed line in (a) was measured by AFM, revealing a height of approximately 80 nm. (d) Surface roughness was quantitatively analyzed along both the horizontal (black dashed line) and vertical (red dashed line) directions within the Au flake region of dashed line shown in (a), yielding a value of 0.46 nm.

S4. AFM characterization of monolayer NaYF4:Eu^3+^ NPs.

Figure S4. (a) A monolayer of NaYF_4_:Eu^3+^ NPs was deposited on the Au flake surface using spin-coating, with AFM characterization revealing morphological features of the NPs. The scale bar is 200 nm. (b) The thickness of the monolayer NaYF_4_: Eu^3+^ NPs was quantitatively determined to be 12 nm, as specifically indicated with white-dashed line in (a).

S5. The X-ray diffractometer (XRD) characterization of β - NaYF_4_.

Figure S5. X - ray diffraction (XRD) pattern of the as-synthesized NaYF_4_: Eu^3+^ NPs resembles the β - NaYF_4_ crystal (JCPDS No. 77−2042).

S6. A detailed discussion of the self-interference effect.

Considering the reflection on a single mirror, the normalized radiative decay rates for electric dipole (ED) and magnetic dipole (MD) emissions can be derived through the following formulas[4, 5]:

$\frac{\Gamma^{ED}}{\Gamma_{0}}=\frac{1}{2}Im\left[ \int_{0}^{u_{max}} \left( 2+R_{12}^{s}+R_{12}^{p}\left( 1-2u^{2} \right) \right)\frac{udu}{l_{1}} \right]$	(S-1)

$\frac{\Gamma^{MD}}{\Gamma_{0}}=\frac{1}{2}Im\left[ \int_{0}^{u_{max}} \left( 2-R_{12}^{p}-R_{12}^{s}\left( 1-2u^{2} \right) \right)\frac{udu}{l_{1}} \right]$	(S-2)

Where the subscript 1 denotes the plane where the emitter is located, and the subscript 2 denotes the reflecting surface. $R_{12}^{s, p}=r_{12}^{s,p}e^{-2kl_{1}s_{12}}$ represents the electric field reflected from the interface between media 1 and 2 that arrives back at the emitter's location. $r_{12}^{s}=\frac{\left( l_{1}-l_{2} \right)}{\left( l_{1}+l_{2} \right)}$ and $r_{12}^{p}=\frac{\left( \varepsilon_{1}l_{2}-\varepsilon_{2}l_{1} \right)}{\left( \varepsilon_{1}l_{2}+\varepsilon_{2}l_{1} \right)}$ denote the reflection coefficients for s- and p-polarization, respectively. $s_{12}$ represents the distance from the emitter's plane 1 to the reflecting interface 2. $\Gamma_{0}$ represents the radiative decay rate of the emitter in a uniform medium 1 with index $n_{1}$. $u=k_{\parallel}/k$ and $l_{1}=-i\sqrt{\frac{n_{j}^{2}}{n_{1}^{2}}-u^{2}}$ represent the parallel and perpendicular components of the wave vector, normalized to the wave number $k=\frac{2\pi}{\lambda}n_{1}$ of the emitter layer.

When the in-plane component of the normalized wave vector is considered, the following relation holds:

$k_{\parallel}=uk$	(S-3)

When considering the perpendicular component of the normalized wave vector, the following relationship holds:

$l_{1}=-i\sqrt{1-u^{2}}$	(S-4)

$k_{\perp}=\sqrt{k^{2}-\left( k_{\parallel} \right)^{2}}=k\sqrt{1-u^{2}}=ik\cdot\left[ -i\sqrt{1-u^{2}} \right]=ikl_{1}$	(S-5)

The electric field at the emitter's position can be expressed as:

$R_{12}^{s, p}=r_{12}^{s,p}e^{-2kl_{1}s_{12}}=r_{12}^{s,p}e^{2i{k_{\perp}s}_{12}}$	(S-6)

It can be observed that

$\varphi_{r}=arg\left( r_{12}^{s,p} \right)$	(S-7)

represents the reflection phase at the interface, while

$\varphi_{prop}=arg\left( e^{2i{k_{\perp}s}_{12}} \right)=2{k_{\perp}s}_{12}$	(S-8)

represents the propagation phase.

The condition for destructive interference is given by:

$\Phi=\varphi_{r}+\varphi_{prop}=\left( 2m+1 \right)\pi, m=0, \pm1,\pm2,\cdots$	(S-9)

When considering small emission angles or perpendicular emission:

$\theta\approx90^{\circ},cos\theta\approx1$	(S-10)

The propagation phase can be expressed as:

$\varphi_{prop}\approx2{\frac{2\pi}{\lambda}n_{1}s}_{12}={\frac{4\pi}{\lambda}n_{1}s}_{12}$	(S-11)

Simultaneously, according to the boundary conditions of the electric field, it follows that:

$\varphi_{r}=arg\left( r_{12}^{s,p} \right)=\pi$	(S-12)

The following condition must be satisfied:

$\varphi_{prop}=arg\left( e^{2i{k_{\perp}s}_{12}} \right)=2{k_{\perp}s}_{12}=0 or 2\pi$	(S-13)

The condition for destructive interference thus becomes:

$\Phi=\varphi_{r}+\varphi_{prop}=\left( 2m+1 \right)\pi, m=0, \pm1,\pm2,\cdots$	(S-14)

$\varphi_{prop}=\left( 2m+1 \right)\pi-\pi, m=0, \pm1,\pm2,\cdots$	(S-15)

${\frac{4\pi}{\lambda}n_{1}s}_{12}=\left( 2m+1 \right)\pi-\pi, m=0, \pm1,\pm2,\cdots$	(S-16)

Excluding the case where the distance between the emitter and the reflecting interface is 0 ($m=0$). For the first occurrence of destructive interference ($m=1$), the relationship between the distance a from the emitter to the reflecting interface and the emission wavelength λ can be derived as:

$s_{12}=\frac{\lambda}{4n_{1}}$	(S-17)

The variation in propagation phase determines the condition for destructive interference. Theoretically, when the distance between the emitter and the reflecting interface is zero, all ED transitions would undergo perfect destructive interference. However, this scenario does not exist in practice. According to Equation (S-11), when the propagation phase varies slightly, the separation distance also changes minimally, yielding:

$\delta\varphi_{prop}={\frac{4\pi}{\lambda}n_{1}\delta s}_{12}$	(S-18)

Equation (S18) demonstrates that for ED transitions, the phenomenon of destructive interference becomes more pronounced as the emission wavelength decreases. As indicated in the article, compared to that of ^5^D_0_ → ^7^F_2_, the variation in ^5^D_0_ → ^7^F_4_ remains largely unchanged. The type of dipole determines whether its self-interference effect is predominantly governed by the electric or magnetic field. It should be noted that Equation (S-2) can be derived by replacing $R_{12}^{s}$ with ${-R}_{12}^{p}$ in Equation (S-1), a transformation that incorporates the π-phase shift and polarization difference between the reflected electric and magnetic fields. Consequently, at wavelengths and distances where ED emission is suppressed, MD emission is typically enhanced, a relationship that is corroborated by the results presented in Figure 2.

S7. SEM characterization of the Au NSs.

Figure S7. (a) SEM characterization of the dispersion density of Au NSs on the Au flake. The scale bar is 4 μm (b) Higher-resolution SEM morphological characterization of individual Au NSs. The scale bar is 1 μm.

S8. The emission spectra of monolayer NaYF4:Eu^3+^ NPs deposited on Si substrate.

Figure S8. The emission spectra of monolayer NaYF_4_:Eu^3+^ NPs deposited on Si substrate were compared between areas with (b) and without (a) individual Au NS. Nearly identical spectral profiles were observed in both configurations, demonstrating that significant modification of the emission characteristics was not achieved by isolated NS alone.

S9. Electromagnetic field distribution of a single Au NS on a Si substrate versus an isolated Au NS.

Figure S9. Panels (a) and (b) represent the electric and magnetic field distributions in the x-z plane of an Au NS on a Si substrate, respectively, while panels (c) and (d) depict the corresponding field distributions in the absence of a substrate.

S10. Magnetic field distribution of a single Au NS at different wavelengths.

In Figure S10, (a-d) represent the electric field distributions of the structure at the respective wavelengths, while (e-h) represent the corresponding magnetic field distributions. As shown in Figure S10(a), the electric field distribution at 532 nm exhibits the highest intensity. While the electric field at 590 nm is also stronger than that at 610 nm, the difference between the two is not substantial. The electric field intensity at 690 nm is the weakest compared to the other wavelengths studied. The strongest localized magnetic field enhancement observed at 590 nm originates from a higher closed-loop displacement current at this wavelength compared to others in Figure S10(f). The resulting oscillating magnetic dipole moment leads to significant magnetic field localization.

Although a single Au NS can alter the magnetic field intensity in its vicinity, this change is relatively weak compared to the significant electric field enhancement. Consequently, it does not effectively modulate the emission of Eu^3+^ ions. As demonstrated in Figure S10, the enhancement effect induced by a single Au NS is primarily attributed to excitation enhancement.

Figure S10. Panels (a-d) represent the simulated electric field distributions in the x-z plane at wavelengths of 532, 590, 610, and 690 nm, respectively. Panels (e-h) show the corresponding magnetic field distribution simulations.

S11. The attenuation of self-interference enhancement effects for magnetic dipole (MD) emissions was observed in multilayer NP systems.

Figure S11. The emission spectra of non-uniform multilayers (>3 layers) of NaYF_4_:Eu^3+^ NPs were characterized on both ITO substrates and Au flake. When the NaYF_4_:Eu^3+^ NP films no longer maintained strict monolayer uniformity, ideal self-interference effects were not achieved within the excitation spot area. This phenomenon was found to prevent interference enhancement of the MD transition emission from Eu^3+^ ions at the metal mirror surface. As a result, the spectral characteristics were observed to be nearly identical between the Au flake and ITO substrate configurations.

S12. Scattering spectrum of a single Au NS.

The scattering intensity of an Au NS can typically be described using the Rayleigh scattering formula[6, 7]:

$I_{s}=I_{0}\frac{1+{cos}^{2} \theta}{2R^{2}}\left( \frac{2\pi}{\lambda} \right)^{4}\left( \frac{n^{2}-1}{n^{2}+2} \right)^{2}r^{6}$	(S-19)

Where $I_{0}$ is the intensity of the incident light, $r$ is the distance from the observer to the particle, $\theta$ is the scattering angle, a is the radius of the Au NS, $\lambda$ is the wavelength of the scattered light, and $n$ is the refractive index of the surrounding medium.

According to Equation (S-19), the intensity of the scattered light is proportional to the sixth power of the Au NS 's radius ($I_{s}\propto r^{6}$). Furthermore, for small Au NSs, the extinction is predominantly governed by absorption.[8, 9] This results in weak scattering signals from small Au NS that are difficult to detect, due to the dissipation and absorption of light by the ITO and Au flake substrates. In Figure S12, the simulated scattering spectrum of a single 60 nm diameter Au NS on an Au flake is provided to demonstrate that the resonant peak of this system does not align with the emission of Eu^3+^ ions.

Figure S12. The simulated dark-field scattering spectrum of a 60 nm diameter Au NS.

S13. The interparticle spacing of Au NSs.

Figure S13. The interparticle spacing of Au NS dimers was systematically characterized through SEM. As shown in panels (a)-(d), four distinct configurations with progressively reduced interparticle distances were examined, where panel (d) specifically demonstrated the direct contact configuration (zero spacing). The scale bars were calibrated at 50 nm for (a) and 100 nm for (b)-(d) respectively. (d) The interparticle spacing of Au NS dimers was precisely determined through high-resolution SEM characterization in Figure 4, with the measured gap distance being quantified as 2.011 nm. To mitigate electron beam-induced damage during SEM characterization, the measurements were performed on separate but structurally equivalent samples. The NS spacings were precisely determined through correlated AFM and SEM analysis.

S14. Branching ratio profiles for the different transitions of MD and ED.

Figure S14. (a) Four-level model of Eu^3+^ ions. (b) Branching ratios(590 nm and 610 nm) of the most important Eu^3+^ emission lines, as a function of the gap distance between the Au NS dimers.

S15. Magnetic field distributions of Au NS dimers with different interparticle gaps.

As seen in Figures S14(a-c), the distributions of the magnetic and electric fields are complementary: when the electric field is enhanced, the magnetic field is correspondingly suppressed in the normal direction.

Since the ED and MD transitions of Eu^3+^ ions share the same excited state (^5^D_0_), their emission processes inherently involve branching ratio competition. As the gap decreases, the structure provides "asymmetric electromagnetic excitation" (electric field enhancement >> magnetic field enhancement), selectively and significantly increasing the excitation efficiency of the ED channel. Within this framework, the ED transition achieves enhancement due to its excitation advantage. Conversely, the MD transition exhibits limited excitation gain and becomes disadvantaged in the radiative branching competition, leading to emission suppression. The concurrent variations in the electric and magnetic fields provide a deeper explanation for the contrasting trends observed in the ED and MD emissions of Eu^3+^ ions.

Figure S15. The magnetic field distributions in the x-z plane were simulated under 532 nm excitation for three configurations: (a) a single Au NS, (b) an Au NS dimer with 20 nm spacing, and (c) an Au NS dimer with 2 nm spacing.

S16. The emission spectra of monolayer NaYF_4_:Eu^3+^ NPs deposited on a Si substrate were systematically investigated under the influence of Au NSs.

Figure S16. The fluorescence emission of NaYF_4_:Eu^3+^ NPs was investigated at both single Au NS and Au NS dimer sites on a Si substrate. (a) The surface morphology of an isolated Au NS on the silicon substrate was characterized by AFM. (d) The corresponding emission spectrum of NaYF_4_:Eu^3+^ NPs was recorded at this location. (b) and (c) The topographic features of different Au NS dimers on the silicon substrate were examined by AFM. The scale bar is 200 nm. (e) and (f) The emission spectra of NaYF_4_:Eu^3+^ NPs were measured at the respective dimer locations. Through comparative analysis, it was determined that the Au NSs exhibited no measurable influence on either the electric dipole (ED, ^5^D_0_→^7^F_2_) or MD (^5^D_0_→^7^F_1_) transition emissions of NaYF_4_:Eu^3+^ NPs when deposited on Si substrates.

S17. The amplitude and SEM images of three Au NS pairs.

Figure S17. (a) AFM Characterization of Au NSs in Figure 5. (b) Amplitude image was referred to the identical positions as presented in (a). The residual traces adjacent to the Au NSs were generated during the nanomanipulation process. (c) Three Au NS dimers were characterized in situ by SEM. The scale bar is 200 nm.

S18. The influence of varying Au NS densities on the emission from monolayer NaYF_4_:Eu^3+^ NPs.

Figure S18. The variations in the emission of Eu^3+^ ion MD transitions and ED transitions under the effect of different quantities of Au NSs were investigated. The emission of MD transitions was continuously weakened, whereas that of ED transitions was progressively enhanced.

Reference

[1]	Z. Li and Y. Zhang, "An efficient and user-friendly method for the synthesis of hexagonal-phase NaYF_4_:Yb, Er/Tm nanocrystals with controllable shape and upconversion fluorescence," *Nanotechnology,* vol. 19, no. 34, p. 345606, 2008/07/16 2008.

[2]	Q. Ruan, L. Shao, Y. Shu, J. Wang, and H. Wu, "Growth of Monodisperse Gold Nanospheres with Diameters from 20 nm to 220 nm and Their Core/Satellite Nanostructures," *Advanced Optical Materials,* vol. 2, no. 1, pp. 65-73, 2013.

[3]	K. Caixia *et al.*, "Gold microplates with well-defined shapes," *Small,* vol. 6, no. 16, pp. 1768-75, Aug 16 2010.

[4]	S. Karaveli and R. Zia, "Spectral tuning by selective enhancement of electric and magnetic dipole emission," *Phys Rev Lett,* vol. 106, no. 19, p. 193004, May 13 2011.

[5]	R. R. Chance, A. Prock, and R. Silbey, *Molecular Fluorescence and Energy Transfer Near Interfaces* (Advances in Chemical Physics). 1978, pp. 1-65.

[6]	S. N. P. John H. Seinfeld, *Atmospheric Chemistry and Physics - From Air Pollution to Climate Change* Wiley, 2006.

[7]	A. J. Cox, A. J. DeWeerd, and J. Linden, "An experiment to measure Mie and Rayleigh total scattering cross sections," *American Journal of Physics,* vol. 70, no. 6, pp. 620-625, 2002.

[8]	E. G. Wrigglesworth and J. H. Johnston, "Mie theory and the dichroic effect for spherical gold nanoparticles: an experimental approach," *Nanoscale Adv,* vol. 3, no. 12, pp. 3530-3536, Jun 15 2021.

[9]	P. K. Jain, K. S. Lee, I. H. El-Sayed, and M. A. El-Sayed, "Calculated Absorption and Scattering Properties of Gold Nanoparticles of Different Size, Shape, and Composition:  Applications in Biological Imaging and Biomedicine," *The Journal of Physical Chemistry B,* vol. 110, no. 14, pp. 7238-7248, 2006/04/01 2006.
